# 1-Benzoyl-3,3-dibutyl­thio­urea

**DOI:** 10.1107/S1600536810037177

**Published:** 2010-09-25

**Authors:** N. Gunasekaran, R. Karvembu, Seik Weng Ng, Edward R. T. Tiekink

**Affiliations:** a Department of Chemistry, National Institute of Technology, Tiruchirappalli 620 015, India; b Department of Chemistry, University of Malaya, 50603 Kuala Lumpur, Malaysia

## Abstract

The title mol­ecule, C_16_H_24_N_2_OS, is twisted about the central N(H)—C bond with a C—N(H)—C—N torsion angle of −62.67 (15)°. The carbonyl group is twisted out of the plane of the benzene ring, forming a C—C—C=O torsion angle of −25.06 (17)°. In the crystal, mol­ecules related by centres of symmetry are linked by pairs of inter­molecular N—H⋯S hydrogen bonds, forming eight-membered {⋯HNCS}_2_ synthons. These are further connected by weak *via* C—H⋯O contacts, forming a two-dimensional array in the *bc* plane.

## Related literature

For pharmaceutical applications of thio­urea derivatives, see: Binzet *et al.* (2009 ▶); Lipowska *et al.* (1996 ▶). For the coordination potential of thio­urea derivatives, see: Henderson *et al.* (2002 ▶); Hallale *et al.* (2005 ▶). For the use of ruthenium(III) complexes of thio­ureas as catalysts, see: Gunasekaran & Karvembu (2010 ▶). For related structures, see: Gunasekaran *et al.* (2010*a* ▶,*b* ▶).
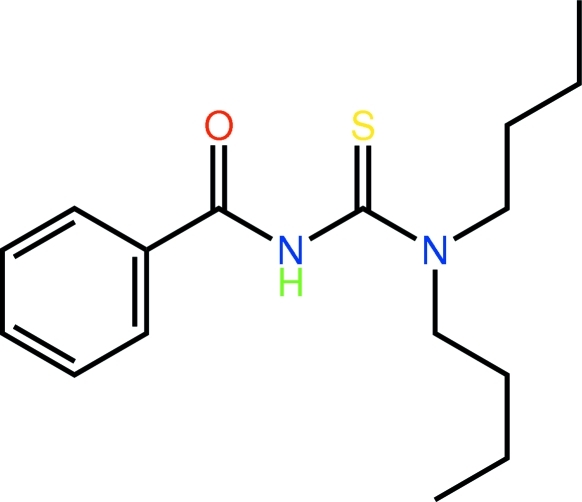


## Experimental

### 

#### Crystal data

C_16_H_24_N_2_OS
*M* *_r_* = 292.43
Monoclinic, 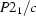

*a* = 10.3213 (7) Å
*b* = 15.7043 (11) Å
*c* = 10.0992 (7) Å
β = 98.751 (1)°
*V* = 1617.91 (19) Å^3^
*Z* = 4
Mo *K*α radiation
μ = 0.20 mm^−1^
*T* = 100 K
0.40 × 0.40 × 0.15 mm

#### Data collection

Bruker SMART APEX diffractometer
Absorption correction: multi-scan (*SADABS*; Sheldrick, 1996 ▶) *T*_min_ = 0.925, *T*_max_ = 0.971
15120 measured reflections
3725 independent reflections
3204 reflections with *I* > 2σ(*I*)
*R*_int_ = 0.036

#### Refinement

*R*[*F* ^2^ > 2σ(*F* ^2^)] = 0.034
*wR*(*F* ^2^) = 0.092
*S* = 1.03
3725 reflections
185 parameters
1 restraint
H atoms treated by a mixture of independent and constrained refinement
Δρ_max_ = 0.31 e Å^−3^
Δρ_min_ = −0.26 e Å^−3^

### 

Data collection: *APEX2* (Bruker, 2008 ▶); cell refinement: *SAINT* (Bruker, 2008 ▶); data reduction: *SAINT*; program(s) used to solve structure: *SHELXS97* (Sheldrick, 2008 ▶); program(s) used to refine structure: *SHELXL97* (Sheldrick, 2008 ▶); molecular graphics: *ORTEP-3* (Farrugia, 1997 ▶) and *DIAMOND* (Brandenburg, 2006 ▶); software used to prepare material for publication: *publCIF* (Westrip, 2010 ▶).

## Supplementary Material

Crystal structure: contains datablocks global, I. DOI: 10.1107/S1600536810037177/lh5132sup1.cif

Structure factors: contains datablocks I. DOI: 10.1107/S1600536810037177/lh5132Isup2.hkl

Additional supplementary materials:  crystallographic information; 3D view; checkCIF report

## Figures and Tables

**Table 1:** Hydrogen-bond geometry (Å, °)

*D*—H⋯*A*	*D*—H	H⋯*A*	*D*⋯*A*	*D*—H⋯*A*
N1—H1*n*⋯S1^i^	0.85 (1)	2.64 (1)	3.4547 (11)	160 (1)
C2—H2a⋯O1^ii^	0.95	2.47	3.4102 (16)	173
C14—H14b⋯O1^iii^	0.99	2.58	3.3559 (16)	136

Symmetry codes: (i) 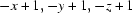; (ii) 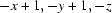; (iii) 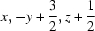.

**Fig. 1.**
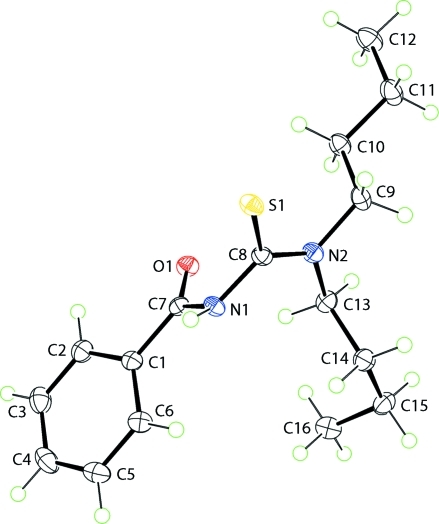
The molecular structure of (I) showing the atom-labelling scheme and displacement ellipsoids at the 50% probability level.

**Fig. 2.**
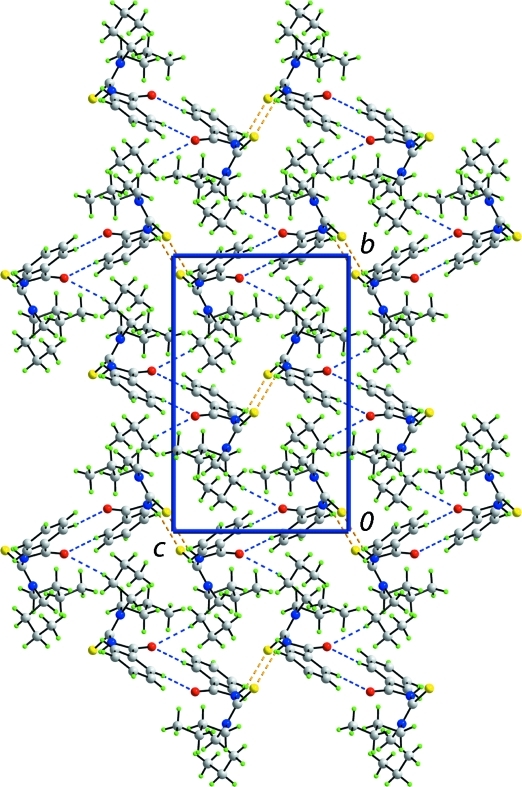
View of the 2-D array in (I). The N–H···S hydrogen bonding and C–H···O contacts are shown as orange and blue dashed lines, respectively.

**Fig. 3.**
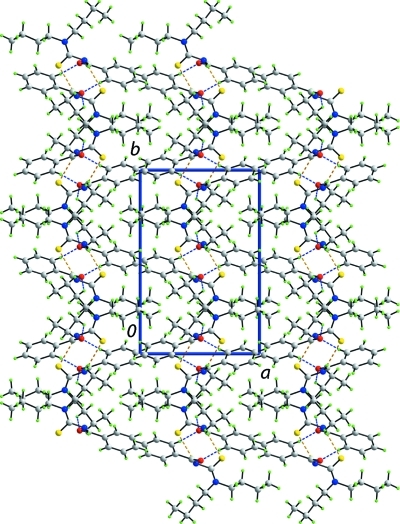
Unit-cell contents shown in projection down the a axis in (I) showing the stacking of layers. The N–H···S hydrogen bonding and C–H···O contacts are shown as orange and blue dashed lines, respectively.

## supplementary crystallographic information

### Comment

Thiourea and its derivatives have important pharmaceutical applications (Binzet *et al.*, 2009; Lipowska *et al.*, 1996). These species are also considered as versatile and attractive ligands due to their coordination ability to a wide range of metal centres, either as neutral ligands, or as mono- or di-anions (Henderson *et al.*, 2002; Hallale *et al.*, 2005). Their coordination complexes can also exhibit useful properties. As an example of a recent application, ruthenium(III) complexes containing these ligands have recently been used as catalysts for oxidation of alcohols to carbonyl compounds (Gunasekaran & Karvembu, 2010). In continuation of structural studies of these molecules (Gunasekaran *et al.*, 2010*a*; Gunasekaran *et al.*, 2010*b*), (I), the crystal structure of the title compound was carried out.

In (I), the molecule is twisted about the central N1—C8 bond as reflected in the value of the C7—N1—C8—S1 torsion angle of 119.81 (11) ° and C7—N1—C8—-N2 of -62.67 (15) °  (see Fig. 1). The carbonyl group is twisted out of the plane of the benzene ring to which it is attached [the C2—C1—C7—O1 dihedral angle = -25.06 (17) °], and the butyl groups lie on opposite sides of the mean plane formed by the N_2_S atoms.

The most prominent intermolecular interactions are of the type N—H···S, occurring between centrosymmetrically related molecules to form an eight-membered {···HNCS}_2_ synthon, Table 1. The dimeric aggregates are linked into a 2-D array *via* C—H···O contacts, Fig. 2 and Table 1. The layers thus formed stack along the *a* axis, Fig. 3.

### Experimental

A solution of benzoyl chloride (0.7029 g, 5 mmol) in acetone (50 ml) was added drop wise to a suspension of potassium thiocyanate (0.4859 g, 5 mmol) in anhydrous acetone (50 ml). The reaction mixture was heated under reflux for 45 min and then cooled to room temperature. A solution of dibutyl amine (0.6462 g, 5 mmol) in acetone (30 ml) was added and the resulting mixture was stirred for 2 h. Hydrochloric acid (0.1 N, 300 ml) was added and the resulting white solid was filtered, washed with water and dried *in vacuo*. Single crystals of (I) for X-ray diffraction were grown at room temperature from its acetone solution. *M*. pt. 358–360 K; Yield 76%. FT—IR (KBr) ν(N—H) 3174, ν(C═O) 1688, ν(C═S) 1243 cm^-1^.

### Refinement

Carbon-bound H-atoms were placed in calculated positions (C—H 0.95 to 0.99 Å) and were included in the refinement in the riding model approximation, with *U*_iso_(H) set to 1.2 to 1.5*U*_equiv_(C). The N-bound H-atom was located in a difference Fourier map, and was refined with a distance restraint of N–H 0.86±0.01 Å; the *U_iso_* value was freely refined.

### Crystal data

**Table:**

C_16_H_24_N_2_OS	*F*(000) = 632
*M**_r_* = 292.43	*D*_x_ = 1.201 Mg m^−^^3^
Monoclinic, *P*2_1_/*c*	Mo *K*α radiation, λ = 0.71073 Å
Hall symbol: -P 2ybc	Cell parameters from 5696 reflections
*a* = 10.3213 (7) Å	θ = 4.4–28.3°
*b* = 15.7043 (11) Å	µ = 0.20 mm^−^^1^
*c* = 10.0992 (7) Å	*T* = 100 K
β = 98.751 (1)°	Block, colourless
*V* = 1617.91 (19) Å^3^	0.40 × 0.40 × 0.15 mm
*Z* = 4	

### Data collection

**Table:**

Bruker SMART APEX diffractometer	3725 independent reflections
Radiation source: fine-focus sealed tube	3204 reflections with *I* > 2σ(*I*)
graphite	*R*_int_ = 0.036
ω scans	θ_max_ = 27.5°, θ_min_ = 2.0°
Absorption correction: multi-scan (*SADABS*; Sheldrick, 1996)	*h* = −12→13
*T*_min_ = 0.925, *T*_max_ = 0.971	*k* = −20→20
15120 measured reflections	*l* = −13→12

### Refinement

**Table:**

Refinement on *F*^2^	Primary atom site location: structure-invariant direct methods
Least-squares matrix: full	Secondary atom site location: difference Fourier map
*R*[*F*^2^ > 2σ(*F*^2^)] = 0.034	Hydrogen site location: inferred from neighbouring sites
*wR*(*F*^2^) = 0.092	H atoms treated by a mixture of independent and constrained refinement
*S* = 1.03	*w* = 1/[σ^2^(*F*_o_^2^) + (0.0441*P*)^2^ + 0.602*P*] where *P* = (*F*_o_^2^ + 2*F*_c_^2^)/3
3725 reflections	(Δ/σ)_max_ = 0.001
185 parameters	Δρ_max_ = 0.31 e Å^−^^3^
1 restraint	Δρ_min_ = −0.26 e Å^−^^3^

### Special details

**Table:**

Geometry. All e.s.d.'s (except the e.s.d. in the dihedral angle between two l.s. planes) are estimated using the full covariance matrix. The cell e.s.d.'s are taken into account individually in the estimation of e.s.d.'s in distances, angles and torsion angles; correlations between e.s.d.'s in cell parameters are only used when they are defined by crystal symmetry. An approximate (isotropic) treatment of cell e.s.d.'s is used for estimating e.s.d.'s involving l.s. planes.
Refinement. Refinement of *F*^2^ against ALL reflections. The weighted *R*-factor *wR* and goodness of fit *S* are based on *F*^2^, conventional *R*-factors *R* are based on *F*, with *F* set to zero for negative *F*^2^. The threshold expression of *F*^2^ > σ(*F*^2^) is used only for calculating *R*-factors(gt) *etc*. and is not relevant to the choice of reflections for refinement. *R*-factors based on *F*^2^ are statistically about twice as large as those based on *F*, and *R*- factors based on ALL data will be even larger.

### Fractional atomic coordinates and isotropic or equivalent isotropic displacement parameters (Å^2^)

**Table:**

	*x*	*y*	*z*	*U*_iso_*/*U*_eq_	
S1	0.31557 (3)	0.56922 (2)	0.46025 (3)	0.01948 (10)	
O1	0.49254 (9)	0.58166 (6)	0.12400 (9)	0.0197 (2)	
N1	0.53208 (10)	0.59664 (7)	0.35231 (10)	0.0161 (2)	
H1n	0.5663 (15)	0.5638 (9)	0.4155 (13)	0.027 (4)*	
N2	0.36726 (10)	0.69784 (7)	0.30300 (10)	0.0178 (2)	
C1	0.70516 (12)	0.54489 (8)	0.23442 (12)	0.0169 (2)	
C2	0.73420 (13)	0.49345 (8)	0.13018 (13)	0.0214 (3)	
H2A	0.6667	0.4767	0.0604	0.026*	
C3	0.86217 (14)	0.46701 (9)	0.12914 (15)	0.0275 (3)	
H3A	0.8821	0.4311	0.0592	0.033*	
C4	0.96133 (14)	0.49266 (9)	0.22958 (15)	0.0281 (3)	
H4A	1.0489	0.4746	0.2278	0.034*	
C5	0.93326 (13)	0.54464 (9)	0.33254 (14)	0.0252 (3)	
H5A	1.0014	0.5624	0.4010	0.030*	
C6	0.80499 (13)	0.57064 (8)	0.33523 (13)	0.0205 (3)	
H6A	0.7853	0.6060	0.4059	0.025*	
C7	0.56729 (12)	0.57488 (7)	0.22861 (12)	0.0159 (2)	
C8	0.40510 (12)	0.62549 (8)	0.36472 (12)	0.0164 (2)	
C9	0.23215 (13)	0.72865 (8)	0.29594 (13)	0.0205 (3)	
H9A	0.2320	0.7916	0.2996	0.025*	
H9B	0.1944	0.7070	0.3739	0.025*	
C10	0.14839 (12)	0.69899 (8)	0.16698 (13)	0.0202 (3)	
H10A	0.1403	0.6362	0.1687	0.024*	
H10B	0.1926	0.7144	0.0899	0.024*	
C11	0.01211 (14)	0.73842 (9)	0.14782 (14)	0.0274 (3)	
H11A	−0.0298	0.7261	0.2276	0.033*	
H11B	0.0202	0.8010	0.1407	0.033*	
C12	−0.07515 (14)	0.70524 (10)	0.02386 (14)	0.0282 (3)	
H12A	−0.1617	0.7321	0.0166	0.042*	
H12B	−0.0845	0.6434	0.0308	0.042*	
H12C	−0.0356	0.7189	−0.0558	0.042*	
C13	0.45409 (13)	0.75523 (8)	0.24042 (12)	0.0194 (3)	
H13A	0.4005	0.7903	0.1711	0.023*	
H13B	0.5153	0.7208	0.1959	0.023*	
C14	0.53205 (13)	0.81332 (8)	0.34351 (13)	0.0218 (3)	
H14A	0.5934	0.7786	0.4065	0.026*	
H14B	0.4713	0.8425	0.3956	0.026*	
C15	0.60954 (14)	0.87993 (8)	0.27769 (14)	0.0247 (3)	
H15A	0.5473	0.9163	0.2184	0.030*	
H15B	0.6574	0.9168	0.3483	0.030*	
C16	0.70717 (14)	0.84140 (9)	0.19606 (14)	0.0265 (3)	
H16A	0.7538	0.8871	0.1570	0.040*	
H16B	0.6603	0.8061	0.1242	0.040*	
H16C	0.7703	0.8062	0.2544	0.040*	

### Atomic displacement parameters (Å^2^)

**Table:**

	*U*^11^	*U*^22^	*U*^33^	*U*^12^	*U*^13^	*U*^23^
S1	0.01575 (16)	0.02258 (17)	0.02072 (17)	0.00138 (11)	0.00475 (12)	0.00432 (12)
O1	0.0204 (5)	0.0224 (4)	0.0150 (4)	−0.0002 (3)	−0.0014 (4)	−0.0001 (3)
N1	0.0144 (5)	0.0204 (5)	0.0133 (5)	0.0021 (4)	0.0010 (4)	0.0027 (4)
N2	0.0170 (5)	0.0197 (5)	0.0162 (5)	0.0014 (4)	0.0007 (4)	0.0015 (4)
C1	0.0165 (6)	0.0178 (6)	0.0169 (6)	−0.0016 (4)	0.0042 (5)	0.0036 (4)
C2	0.0238 (7)	0.0202 (6)	0.0207 (6)	−0.0016 (5)	0.0048 (5)	0.0000 (5)
C3	0.0292 (8)	0.0245 (7)	0.0310 (8)	0.0039 (6)	0.0121 (6)	−0.0009 (6)
C4	0.0199 (7)	0.0292 (7)	0.0372 (8)	0.0061 (5)	0.0102 (6)	0.0093 (6)
C5	0.0179 (7)	0.0312 (7)	0.0258 (7)	−0.0025 (5)	0.0010 (5)	0.0064 (6)
C6	0.0191 (6)	0.0247 (6)	0.0178 (6)	−0.0020 (5)	0.0038 (5)	0.0020 (5)
C7	0.0174 (6)	0.0143 (5)	0.0160 (6)	−0.0028 (4)	0.0026 (5)	0.0009 (4)
C8	0.0146 (6)	0.0199 (6)	0.0140 (6)	0.0005 (4)	−0.0001 (4)	−0.0013 (4)
C9	0.0199 (6)	0.0220 (6)	0.0191 (6)	0.0066 (5)	0.0015 (5)	0.0000 (5)
C10	0.0190 (6)	0.0241 (6)	0.0171 (6)	0.0036 (5)	0.0016 (5)	0.0003 (5)
C11	0.0239 (7)	0.0314 (7)	0.0248 (7)	0.0096 (6)	−0.0025 (5)	−0.0028 (6)
C12	0.0210 (7)	0.0386 (8)	0.0238 (7)	0.0056 (6)	−0.0008 (5)	0.0007 (6)
C13	0.0229 (6)	0.0174 (6)	0.0176 (6)	−0.0008 (5)	0.0019 (5)	0.0023 (5)
C14	0.0217 (7)	0.0247 (6)	0.0190 (6)	0.0007 (5)	0.0028 (5)	−0.0045 (5)
C15	0.0270 (7)	0.0216 (6)	0.0247 (7)	−0.0026 (5)	0.0013 (5)	−0.0051 (5)
C16	0.0227 (7)	0.0313 (7)	0.0253 (7)	−0.0010 (6)	0.0029 (6)	0.0025 (6)

### Geometric parameters (Å, °)

**Table:**

S1—C8	1.6843 (13)	C9—H9B	0.9900
O1—C7	1.2144 (15)	C10—C11	1.5221 (18)
N1—C7	1.3955 (16)	C10—H10A	0.9900
N1—C8	1.4102 (16)	C10—H10B	0.9900
N1—H1n	0.854 (9)	C11—C12	1.5188 (19)
N2—C8	1.3259 (16)	C11—H11A	0.9900
N2—C9	1.4675 (16)	C11—H11B	0.9900
N2—C13	1.4790 (16)	C12—H12A	0.9800
C1—C6	1.3938 (18)	C12—H12B	0.9800
C1—C2	1.3953 (18)	C12—H12C	0.9800
C1—C7	1.4915 (17)	C13—C14	1.5194 (17)
C2—C3	1.3861 (19)	C13—H13A	0.9900
C2—H2A	0.9500	C13—H13B	0.9900
C3—C4	1.387 (2)	C14—C15	1.5289 (19)
C3—H3A	0.9500	C14—H14A	0.9900
C4—C5	1.387 (2)	C14—H14B	0.9900
C4—H4A	0.9500	C15—C16	1.5212 (19)
C5—C6	1.3896 (19)	C15—H15A	0.9900
C5—H5A	0.9500	C15—H15B	0.9900
C6—H6A	0.9500	C16—H16A	0.9800
C9—C10	1.5225 (18)	C16—H16B	0.9800
C9—H9A	0.9900	C16—H16C	0.9800
			
C7—N1—C8	122.03 (10)	C9—C10—H10B	109.2
C7—N1—H1n	112.7 (11)	C11—C10—H10B	109.2
C8—N1—H1n	114.3 (11)	H10A—C10—H10B	107.9
C8—N2—C9	121.01 (11)	C12—C11—C10	112.69 (12)
C8—N2—C13	124.66 (11)	C12—C11—H11A	109.1
C9—N2—C13	114.30 (10)	C10—C11—H11A	109.1
C6—C1—C2	119.95 (12)	C12—C11—H11B	109.1
C6—C1—C7	122.14 (11)	C10—C11—H11B	109.1
C2—C1—C7	117.80 (11)	H11A—C11—H11B	107.8
C3—C2—C1	119.57 (13)	C11—C12—H12A	109.5
C3—C2—H2A	120.2	C11—C12—H12B	109.5
C1—C2—H2A	120.2	H12A—C12—H12B	109.5
C4—C3—C2	120.37 (13)	C11—C12—H12C	109.5
C4—C3—H3A	119.8	H12A—C12—H12C	109.5
C2—C3—H3A	119.8	H12B—C12—H12C	109.5
C3—C4—C5	120.29 (13)	N2—C13—C14	111.42 (10)
C3—C4—H4A	119.9	N2—C13—H13A	109.3
C5—C4—H4A	119.9	C14—C13—H13A	109.3
C4—C5—C6	119.73 (13)	N2—C13—H13B	109.3
C4—C5—H5A	120.1	C14—C13—H13B	109.3
C6—C5—H5A	120.1	H13A—C13—H13B	108.0
C5—C6—C1	120.08 (13)	C13—C14—C15	111.74 (11)
C5—C6—H6A	120.0	C13—C14—H14A	109.3
C1—C6—H6A	120.0	C15—C14—H14A	109.3
O1—C7—N1	122.67 (11)	C13—C14—H14B	109.3
O1—C7—C1	122.54 (11)	C15—C14—H14B	109.3
N1—C7—C1	114.77 (10)	H14A—C14—H14B	107.9
N2—C8—N1	116.41 (11)	C16—C15—C14	113.39 (11)
N2—C8—S1	124.81 (10)	C16—C15—H15A	108.9
N1—C8—S1	118.73 (9)	C14—C15—H15A	108.9
N2—C9—C10	110.64 (10)	C16—C15—H15B	108.9
N2—C9—H9A	109.5	C14—C15—H15B	108.9
C10—C9—H9A	109.5	H15A—C15—H15B	107.7
N2—C9—H9B	109.5	C15—C16—H16A	109.5
C10—C9—H9B	109.5	C15—C16—H16B	109.5
H9A—C9—H9B	108.1	H16A—C16—H16B	109.5
C9—C10—C11	112.19 (11)	C15—C16—H16C	109.5
C9—C10—H10A	109.2	H16A—C16—H16C	109.5
C11—C10—H10A	109.2	H16B—C16—H16C	109.5
			
C6—C1—C2—C3	1.20 (19)	C9—N2—C8—N1	172.80 (10)
C7—C1—C2—C3	177.48 (11)	C13—N2—C8—N1	−9.26 (17)
C1—C2—C3—C4	−1.2 (2)	C9—N2—C8—S1	−9.85 (17)
C2—C3—C4—C5	0.4 (2)	C13—N2—C8—S1	168.09 (9)
C3—C4—C5—C6	0.4 (2)	C7—N1—C8—N2	−62.67 (15)
C4—C5—C6—C1	−0.3 (2)	C7—N1—C8—S1	119.81 (11)
C2—C1—C6—C5	−0.46 (19)	C8—N2—C9—C10	−93.34 (14)
C7—C1—C6—C5	−176.57 (12)	C13—N2—C9—C10	88.52 (13)
C8—N1—C7—O1	2.17 (18)	N2—C9—C10—C11	−173.07 (11)
C8—N1—C7—C1	−179.45 (11)	C9—C10—C11—C12	−176.53 (12)
C6—C1—C7—O1	151.14 (12)	C8—N2—C13—C14	−82.29 (15)
C2—C1—C7—O1	−25.06 (17)	C9—N2—C13—C14	95.77 (12)
C6—C1—C7—N1	−27.25 (16)	N2—C13—C14—C15	−173.16 (10)
C2—C1—C7—N1	156.56 (11)	C13—C14—C15—C16	−59.57 (15)

### Hydrogen-bond geometry (Å, °)

**Table:**

*D*—H···*A*	*D*—H	H···*A*	*D*···*A*	*D*—H···*A*
N1—H1n···S1^i^	0.85 (1)	2.64 (1)	3.4547 (11)	160 (1)
C2—H2a···O1^ii^	0.95	2.47	3.4102 (16)	173
C14—H14b···O1^iii^	0.99	2.58	3.3559 (16)	136

Symmetry codes: (i) −*x*+1, −*y*+1, −*z*+1; (ii) −*x*+1, −*y*+1, −*z*; (iii) *x*, −*y*+3/2, *z*+1/2.
